# Clinical and microbial study of reinforced 3D-printed maxillary denture base resin: a randomized trial

**DOI:** 10.1186/s12903-025-06601-2

**Published:** 2025-07-17

**Authors:** Eman Mohamed Ahmed Teama, Hoda Mohamed Amin Rashad, Eman Abd El-salam Mohamed Shakal, Eman Elsayed Hegazy

**Affiliations:** 1 Prosthodontic Department, Faculty of Dentistry, Tanta University, Tanta, Egypt; 2 Medical Microbiology and Immunology Department, Faculty of Medicine, Tanta University, Tanta, Egypt

**Keywords:** 3D-printed maxillary acrylic resin denture base, ZrO_2_ nanoparticles, TiO_2_ nanoparticles, Microbiological colonization, Patient satisfaction

## Abstract

**Statement of problem:**

The 3D-printed denture base resin needed reinforcement.

**Purpose:**

To evaluate the effects of adding nano ZrO2 and nano TiO2 on microbial colonization and patient satisfaction with 3D-printed maxillary complete dentures.

**Materials and methods:**

Twenty-four patients who needed complete dentures were randomly distributed into three equal groups. Group I: Patients used maxillary complete dentures 3D printed without the addition of any additives. Group II: Patients used maxillary complete dentures 3D after reinforcement by Nano-ZrO_2_ (0.4%) by weight. Group III: Patients used maxillary complete dentures 3D printed after reinforcement by Nano-TiO_2_ (0.4%) by weight. For microbial evaluation, a cotton swab was taken from the mucosa of the palate and the intaglio surface of maxillary dentures, and microbial colonization was evaluated by calculating the number of colony-forming units of *S. aureus on* mannitol salt agar plates and *C. albicans* on Sabouraud’s dextrose agar plates after 48 h of incubation at insertion, 6 months, 12 months and 18 months. Patient satisfaction was evaluated 15 days after insertion and at 6, 12, and 18 months. The values of microbial colonization and patient satisfaction were analyzed via repeated-measures ANOVA followed by Tukey’s multiple comparison test.

**Results:**

No *significant differences in microbial colonization were detected among the three groups concerning Staphylococcus aureus* in the palatal mucosa. There was a significant difference between Group I, the lowest antimicrobial group, and the other groups, while between Groups II and III, there was no significant difference *in the number of S. aureus* on the fitting surface of the denture. There were significant differences between Group II, the highest antifungal group, and the other groups at 12 and 18 months *concerning Candida albicans* in the palate and in the dentures. There was a significant difference in patient satisfaction between Group I, the lowest, and the other groups, whereas there was no significant difference between Groups II and III.

**Conclusion:**

Compared with the other groups, the nano-ZrO_2_ group presented greater antimicrobial effects until 18 months, whereas the nano-TiO2 group presented antibacterial effects until 18 months and antifungal effects until 6 months. The addition of nano-ZrO_2_ and nano-TiO_2_ to 3D-printed denture base resin improved the aesthetic, speech, masticatory efficiency, hygiene, and comfort of patients.

**Trial registration:**

The trial was registered in the Clinical Trials Registry under the number NCT06921577 on 10/04/2025 (retrospective registration).

**Supplementary Information:**

The online version contains supplementary material available at 10.1186/s12903-025-06601-2.

## Introduction

Complete denture demand has continuously increased until now. Many studies are still ongoing on increasing the quality of complete dentures and others. Digital technologies play an important role in increasing the quality and serviceability of partial and complete dentures. Digital technologies will lead to great changes in dentistry in the future, especially in terms of treatment time and simplicity [1, 2].

Subtractive [computerized numeric control milling (CNC milling)] and additive (3D printing) manufacturing techniques) The two main systems of computer-aided design—computer-aided manufacturing are used in the manufacturing of complete removable dental prostheses [3]. Milling pre-polymerized blanks are the technique of the milling method used to make dentures, whereas adding materials (composites, metals, and ceramics) layer by layer is the technique of the manufacturing method [4]. 

Several factors affect the results of 3D printing, including the printing orientation, light source, printing layer thickness, and intensity [5]. Therefore, setting printer parameters properly is critical to produce the best outcomes [6]. The type of material used in the fabrication of 3D-printed prostheses under different conditions affects the reproducibility and strength of the prostheses [5]. [7]. 

Specific alterations in the oral microbiota associated with complete denture wear are crucial for developing preventive strategies, improving denture hygiene practices, and enhancing the overall oral health of denture wearers. The wearing of complete dentures alters the oral environment, affecting factors such as salivary flow, oral hygiene practices, and oral microflora colonization. These changes can lead to shifts in the structure, diversity, and balance of the oral microbiota [8]. 

Failure of removable resin prosthesis after clinical use may be due to cracks formation, and microbial colonization [9]. Reinforced 3D-printed resin has superior properties, but the type and concentration of nanoparticles play an important role in improving the properties [10]. 

The mechanical properties of polymethylmethacrylate (PMMA) were improved by the addition of nanoparticles, as they can make heavy bonds, which make PMMA more reactive when associated with other particles. New improvements in nanotechnology, such as silicon dioxide (SiO2), zinc oxide (ZnO), copper oxide (CuO), zirconium dioxide (ZrO2), and titanium dioxide (TiO2), have been developed to improve the clinical performance and the properties of denture-based resins [11, 12].

The addition of ZrO2 NPs has notable antimicrobial properties as they produce active oxygen species, which enhance their antimicrobial effects [13]. Gad et al. [14] study concluded that *Candida albicans* adhesion was lower in ZrO2 samples than in cold acrylic resin samples. The addition of nano-ZrO2 to the material for relining might offer antifungal properties, decreasing the frequency of denture stomatitis, as previously reported [15]. 

Titanium dioxide nanoparticles are of low-cost, biocompatible materials with high chemical stability, low toxicity, high strength, and a high refractive index, and have a wide range of activities against many microorganisms [16]. Moreover, research has shown that the addition of a minor amount of a nanoTiO_2_ strengthening agent to a polymeric material can alter the chemical, optical, physical, and electrical properties of the composite material [17, 18].

Adding 0.4% of nanoparticles to 3D printed denture base resin shows good biocompatibility and excellent structural, electrical, thermal, and specific performances [19]. Also previous clinical study found that reinforcement of 3D printed denture base resin with TiO_2_ 0.4% by wt. was a viable treatment choice for completely edentulous patients [20]. 

This study aimed to compare the effects of adding nanoZrO_2_ (0.4% wt) and nanoTiO_2_ (0.4% wt.) to 3D-printed acrylic resin dentures in completely edentulous patients concerning microbial colonization and patient satisfaction.

## Material and method

### Ethical consideration

Approval for this research was obtained from the Research Ethics Committee, Faculty of Dentistry, Tanta University (RP 2–23 1). The purpose of the present study was explained to the patients, and informed consent was obtained according to the guidelines on human research adopted by the Research Ethics Committee, Faculty of Dentistry, Tanta University. With clinicaltrials.gov.

ID: NCT06921577 on 10/04/2025(retrospective registration).

### Eligibility criteria

The inclusion criteria were completely edentulous patients with good oral and systemic health that have adequate interarch space with class I Angle^’^s classification, good neuromuscular control, and age ranging from 50 to 70 years.

The exclusion criteria were patients with any disease that may affect denture construction, and patients with oral parafunctional habits, or temporomandibular joint disorders.

### Patients allocation

**Fig. 1:**
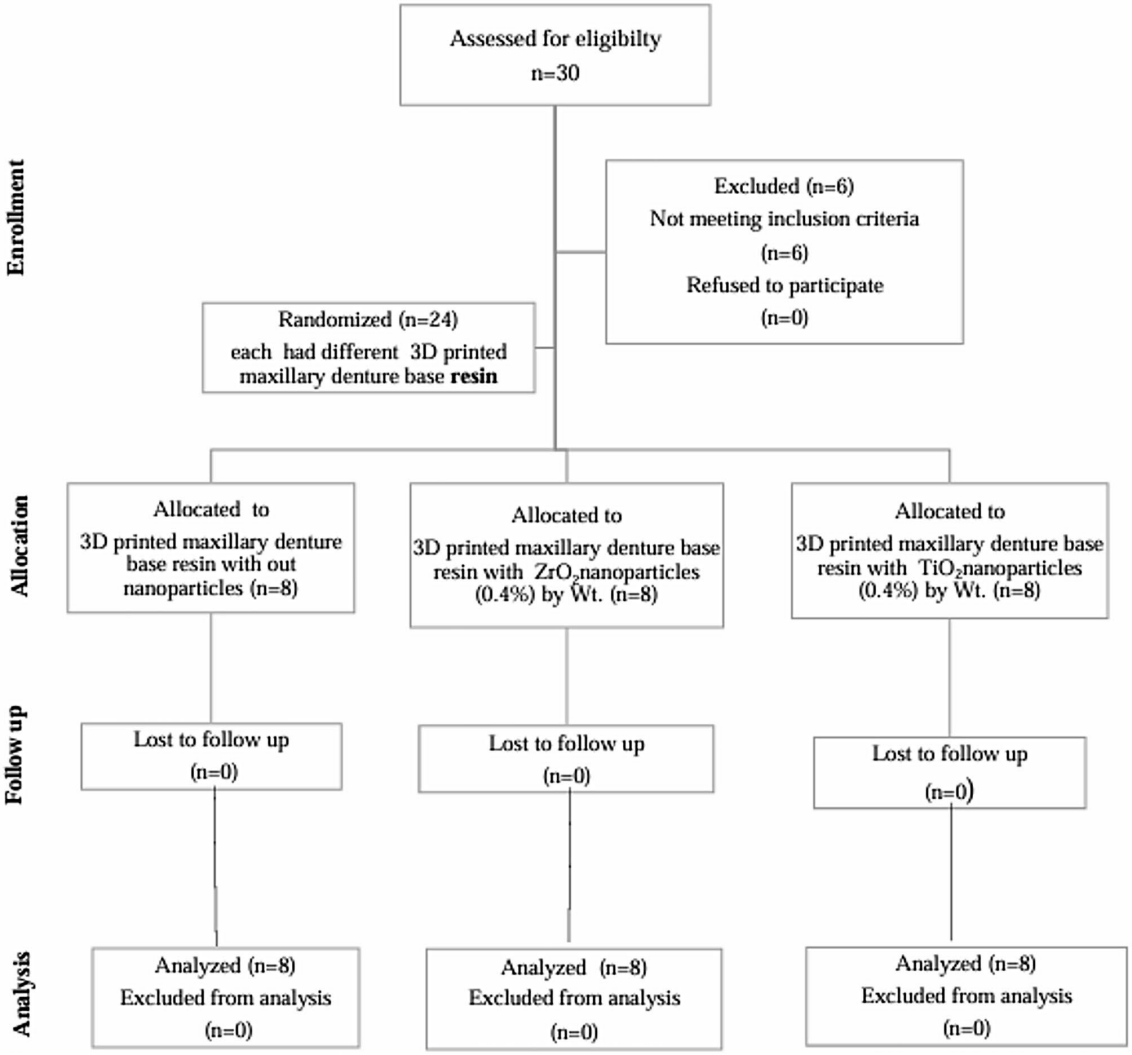
Consort flow diagram

A randomized clinical trial was conducted on twenty-four completely edentulous patients classified in block randomization manner into three equal groups 8 in each: Group I: patients who used maxillary complete dentures 3D printed without additives; Group II: patients who used maxillary complete dentures 3D printed after reinforcement by Nano-ZrO_2_ (0.4%) by Wt; and Group III: patients who used maxillary complete dentures 3D printed after reinforcement by Nano-TiO_2_ (0.4%) by Wt. (Fig. 1). Patients were selected from those who attended the Faculty of Dentistry from the outpatient clinic of the Department of Prosthodontics, Tanta University, Egypt. The sample size was calculated using a computer program, G Power version 3.1.9, depending on the previous study [21]. 

### Preparation of nanoparticles

A Private laboratory (Nanogate Laboratory, Cairo, Egypt^)^ prepared the nanoparticles (Nanogate, Sigma-Aldrich, Germany) with an average size of < 50 nm and in a spherical shape [22]. A high-resolution transmission electron microscope (JEM-2100, Jeol, Akishima, Japan )and X-ray diffraction analysis using a powder diffract meter system(X’pertProPanalytical, Malvern, United Kingdom) were used to confirm the construction of the nanoparticle.

Zirconium dioxide and titanium dioxide nanoparticles were silanized by a silane coupling agent (3-aminopropyltriethoxysilane (APTES)) and then 0.4 g. Of silanized nanoparticles were suspensed in 100 ml of 3D printed resin with sonication and vortexed for 30 min to get a homogenous dispersion.

**The following steps were carried out for all patients**

**Fig. 2:**
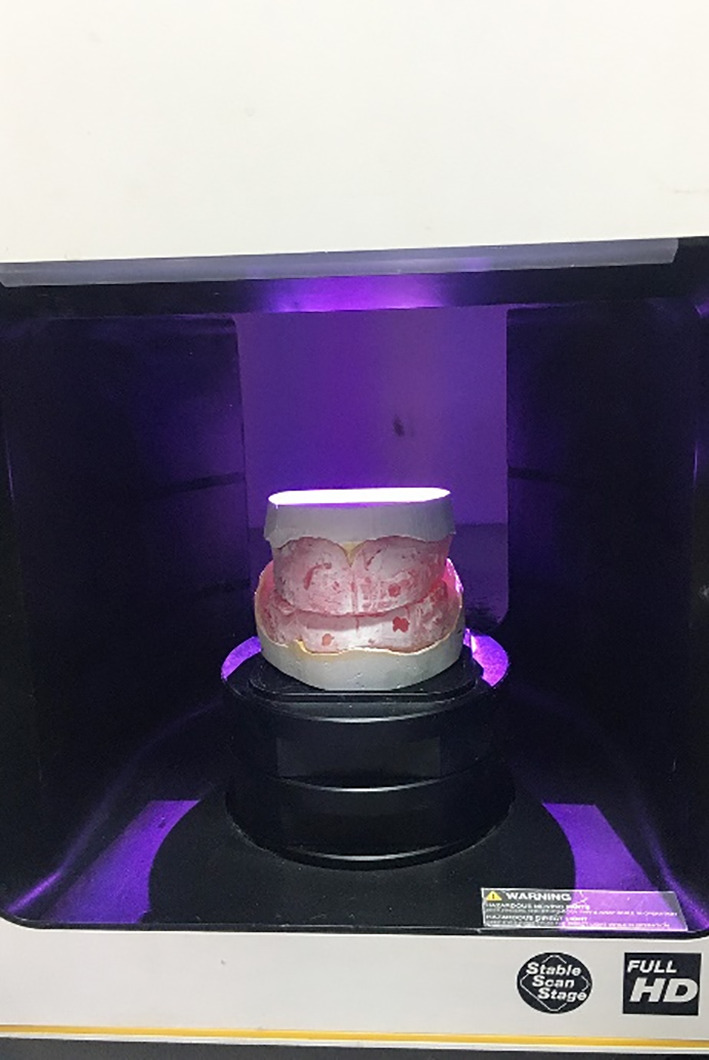
Scanning of record blocks by extraoral scanner

**Fig. 3:**
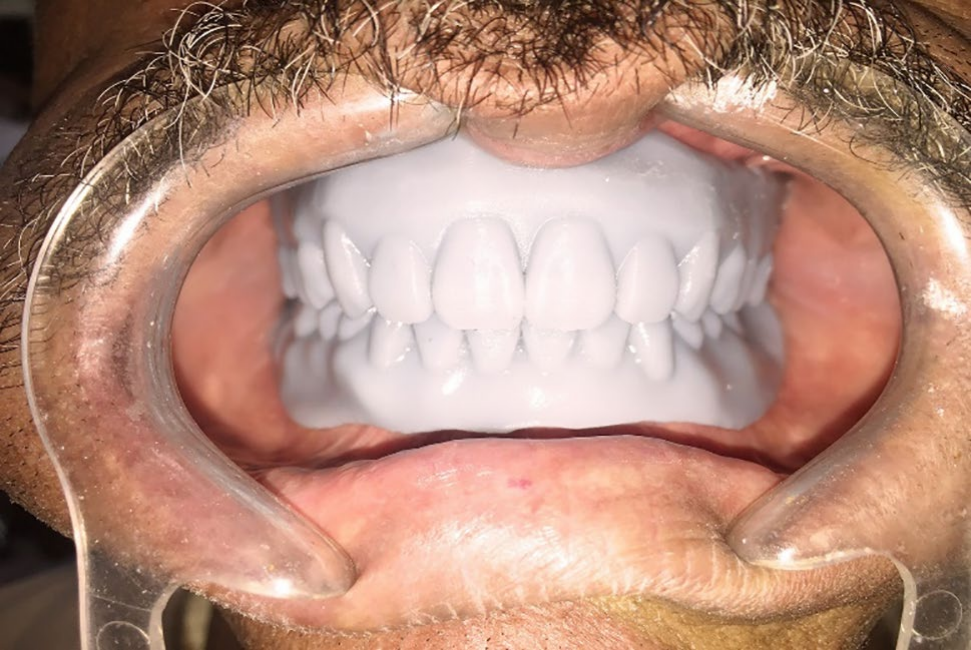
3D printed trial denture base in the patient’s mouth

**Fig. 4:**
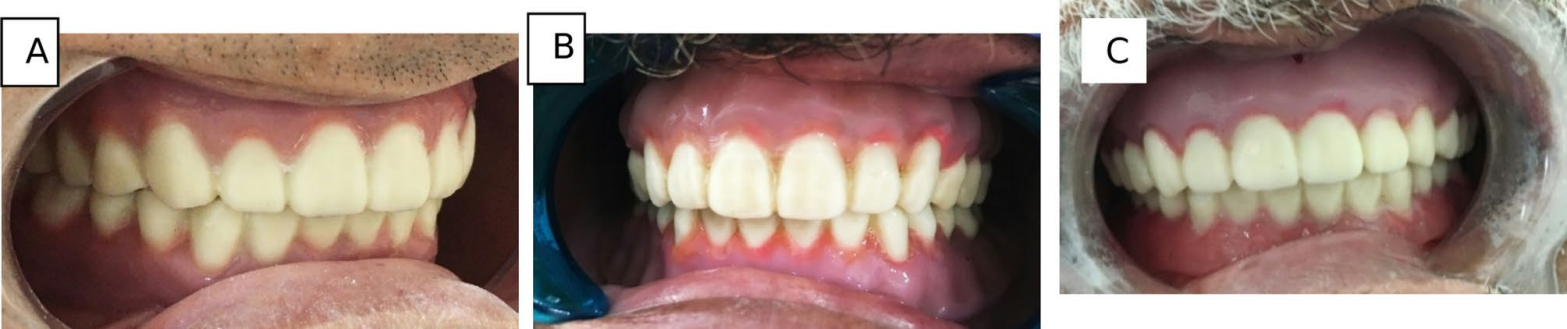
Final denture in the patient’s mouth during insertion, 3D printed denture without nanoparticles (**A**), 3D printed (upper) denture base with ZrO_2_ nanoparticles (**B**), and 3D printed (upper) denture base with TiO_2_ nanoparticles(**C**)

- Primary and final impressions of the maxillary and mandibular arches were taken for each patient following basic prosthetic principles [23].
- Maxillary and mandibular occlusal rims were made on the master casts and adjusted in the oral cavity of the patients in a normal manner to ensure an acceptable vertical dimension at occlusion and a freeway space of 2–4 mm. The centric relationship was recorded via the static method [24].
- An extra-oral scanner (DOF Freedom-HD Laboratory Scanner Unit, Korea) was used for scanning master casts (upper and lower), then occlusion blocks on the master cast were scanned after spraying with scanner spray (YETI Dental product GmbH, Germany) [24] (Fig. 2).
- Master casts and jaw relation records. Standard tessellation language (STL) files were sent to the software platform (Exocad GmbH, Germany). The software enabled virtually simultaneous mounting and alignment.
- Virtual design of the upper and lower bases and virtual setting of the upper and lower teeth were performed.
- The virtual denture STL file was exported to the LCD printer (Mogassam, MicroDent 1 pro-3D printer, India), which prints the denture base and teeth (trial base) from 3D printed try-in resin (Nextdent Co., Tryin TI0, Soesterberg, Netherlands ) as one unit.
- The try-in step was performed via the trial denture base to check for occlusion, esthetics, and denture border extension (Fig. 3).
- If any modification was performed in teeth by using articulating paper or in the denture base by pressure-indicating paste, the trial denture bases were scanned by an extraoral scanner, and an STL file was sent again to the software for correction of the design, and then sent to the 3D printer software.
- Before printing, each file was checked for support adequacy, the printing orientation was 45 degrees, the thickness of the printed layer was 50 microns, and the printing time was 4 h and 7 s for each denture [25].
- The denture base and the teeth were printed separately via a 3D printer [26].
- For Group Ι, Pink denture base resin (Nextdent Co., Soesterberg, Netherlands) was used without nanoparticles for printing the denture bases. However, for Group II, we used Pink denture base resin reinforced by nanoparticles (ZrO2) (0.4%) by weight.
- Additionally, for Group III, we used pink denture base resin reinforced with nanoparticles (TiO2) (0.4%) by weight.
- For printing the teeth, white tooth resin (Nextdent Co., Soesterberg, Netherlands) was used for all groups.
- To remove any residual monomers after printing, the denture bases and teeth were placed in isopropyl alcohol (Piochem, Egypt) for five minutes.
- Finally, we used resin (Pentron Breeze Self Adhesive Resin, United States) to attach teeth to recessed pockets in the denture bases, after which the dentures were placed in the post-curing unit (Bre.lux PowerUnit2, bredent medical GmbH & Co.KG, Senden– Germany).
- Eventually, the dentures in the three groups were similarly finished by using silicon carbide grinding (standardized finishing), and the teeth were coated with resin glaze (Shofu Inc., resin glaze, United States) to protect and polish areas of posterior pits and fissures and interproximal surfaces from staining or discoloration.
- The dentures were inserted into the patient’s mouth (Fig. 4A, B&C,) and checked for any necessary adjustments, and every patient was given the same post-insertion instructions for home care and follow-up evaluation, and a regular oral and denture hygiene protocol.

## Methods of evaluation

### Microbial evaluation [27]

The microbial evaluation was done at four time points before insertion of the denture, and then at 6, 12, and 18 months after insertion.

- All patients were instructed to never use mouthwash or eat any food or drinks for 60 min before the samples were collected. The samples were collected in the morning at 10–12:00.
- Sterilized disposable cotton swabs were used to collect samples by swabbing the mucosa of the palate and the fitting surface of the maxillary dentures (Figs. 5 & 6), after which they were placed in thioglycolate broth (Himedia Laboratories, India.) as a transport medium. Fig. 7.
  **Fig. 5:**
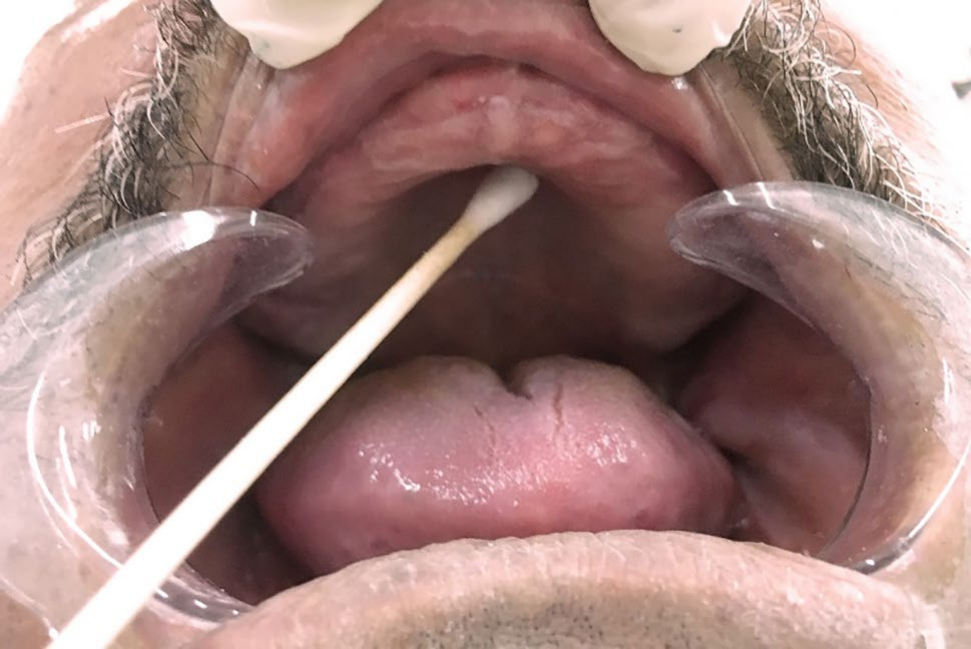
Sample collected from the palate with sterile cotton swab
  **Fig. 6:**
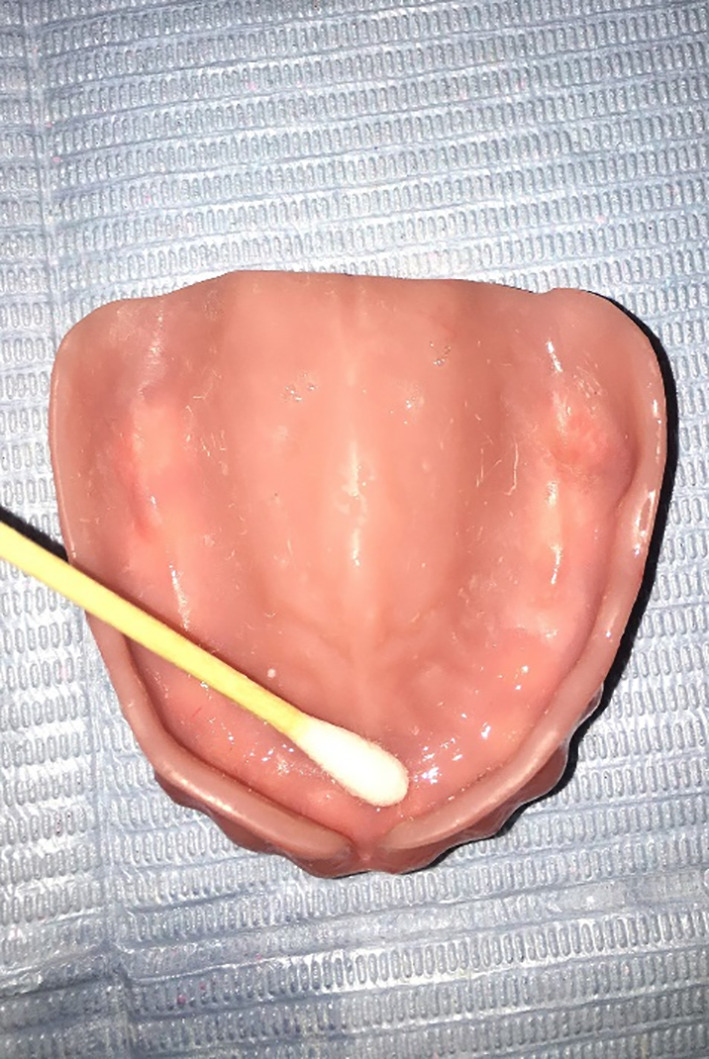
Sample collected from the fitting surface of th e denture with sterile cotton
  **Fig. 7:**
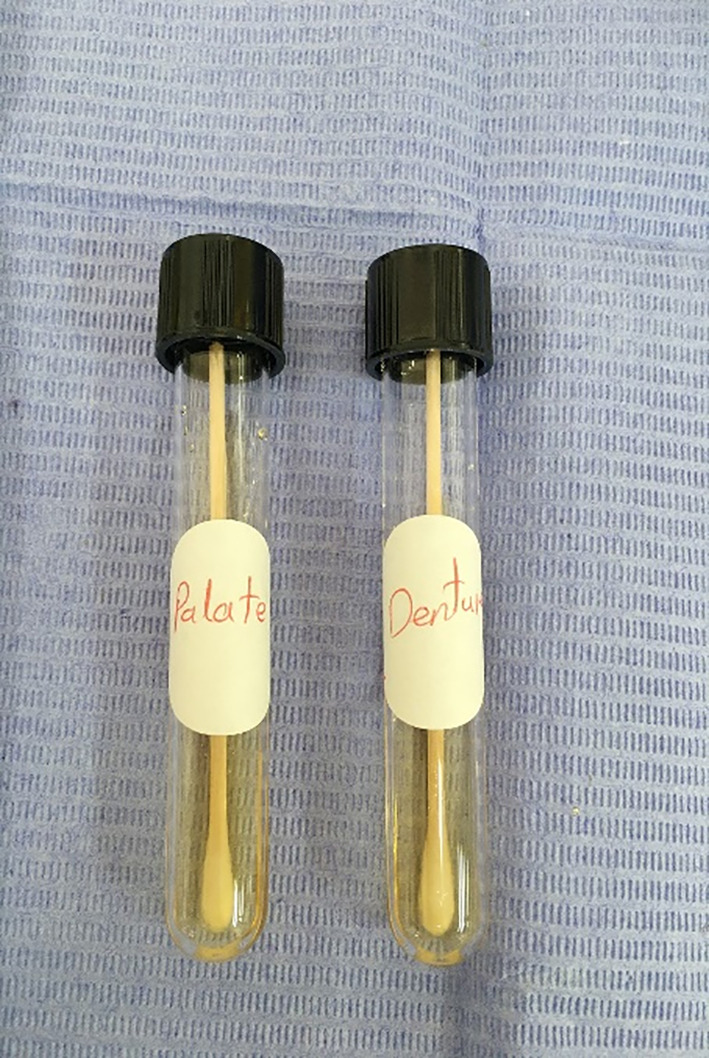
Cotton swab in thioglycolate broth as a transport medium
- Serial 10‑fold dilutions of the transport medium containing the sample were prepared with sterile saline solution up to a dilution factor of 10^‑ [5]^.
- One hundred microliters of the diluted samples were inoculated on Sabouraud’s dextrose agar (Himedia Laboratories, India.) plates for the isolation of *Candida albicans* and on mannitol salt agar (Biomark Laboratories, India) plates that were selective for the isolation of *Staphylococcus aureus.*
- The inoculated plates were subsequently placed in an incubator under aerobic conditions for 48 h of incubation at 37 °C.

**Fig. 8:**
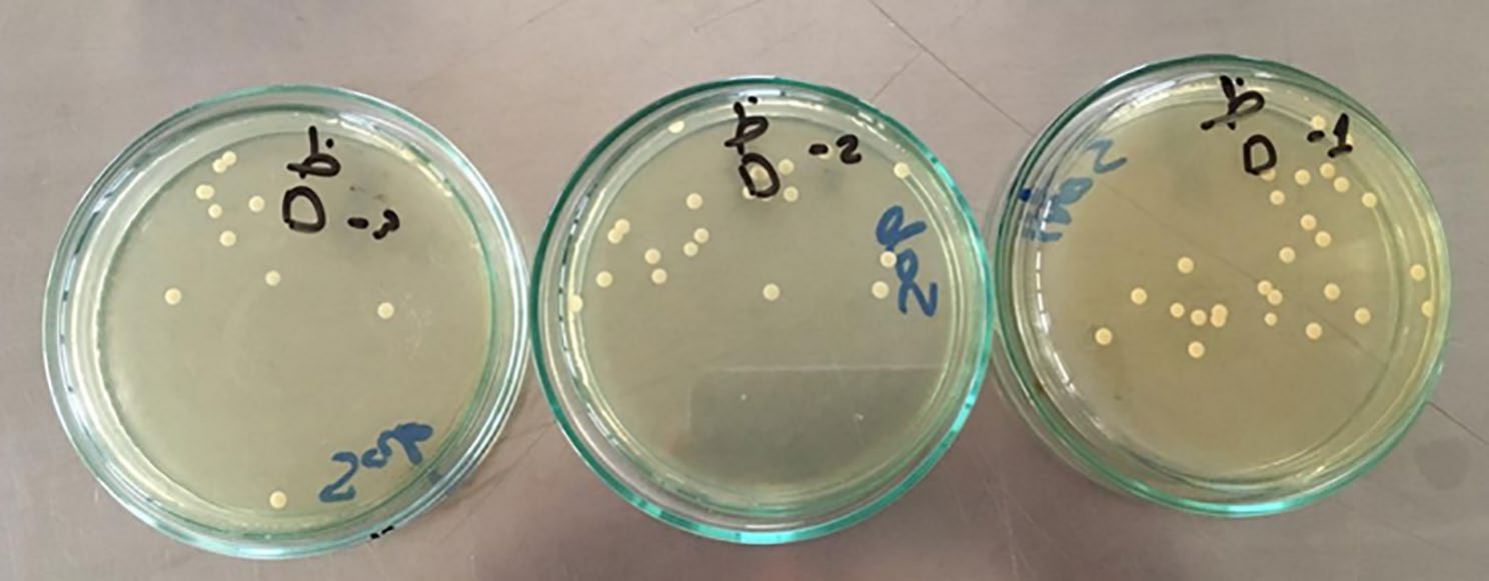
Sabouraud’s dextrose agar plates with fungi growing on it

**Fig. 9:**
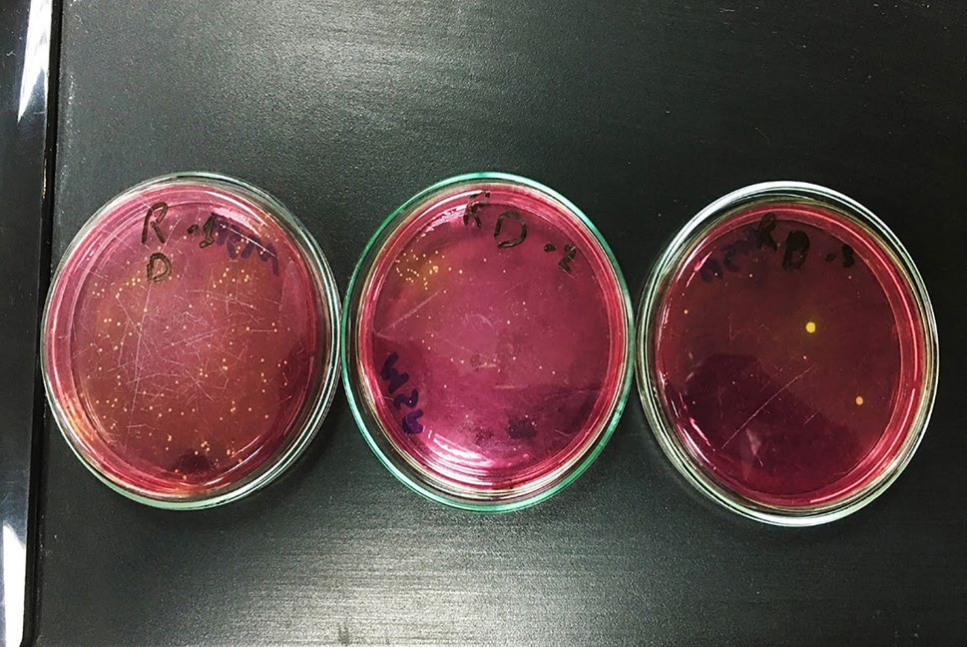
Manitol dextrose agar plates with staphylococcus aurous grow on it

The microbial evaluation was performed by counting colony-forming units (CFUs) of *Staphylococcus aureus* and *Candida albicans* on the surface of the agar plates after 48 h of incubation. Colonies characteristic^’^s of *Staphylococcus aureus* (yellow colonies on mannitol salt agar) and *Candida albicans* (creamy white colonies on Sabouraud’s agar) (Fig. 8 & 9). The CFU count was multiplied by the dilution factor to calculate the number of CFUs per sample. Results were represented as CFU/mL.

### Patient satisfaction

The evaluation was carried out 15 days after insertion to be sure that the patient had adjusted to the denture completely [28] and then after 6, 12, and 18 months.

Patients were requested to evaluate their overall satisfaction with their dentures through the visual analog scale (VAS). This method is a good way to record, quantify, and evaluate qualitative answers that cannot be measured directly [20]. The patients were asked five questions away from the prosthodontist by another colleague, for example, “How satisfied were you with your prosthesis considering aesthetics, speech, masticatory efficiency, hygiene, and comfort?” after translating them into Arabic. A ten-point scale was used to evaluate each question; those who were not satisfied at all took zero points, while those who were completely satisfied took ten points and the data was recorded for each patient throughout follow-up periods to be analyzed statistically [20]. 

### Statistical analysis

The Statistical Package for Social Sciences (SPSS version 22) was used to analyze the collected data statistically. Comparisons among the three groups were performed via analysis of variance (ANOVA). Differences between means greater than Tukey’s post hoc value were considered significant. A significant difference was indicated by a P value < 0.05, and a highly significant difference was indicated by a P value < 0.001.

## Results

### Microbial colonization result

#### Staphylococcus aureus in palatal mucosa

The standard deviation (SD) and mean values of the number of colony-forming units (CFUs) of *Staphylococcus aureus* in the palate throughout the study duration for the studied groups and the comparisons between different durations in each group via repeated-measures ANOVA are shown in (Table 1).

**Table 1:** Analysis of CFU counts of *Staphylococcus aureus* in the palatal mucosa at different observation periods in the three groups

Duration	Staphylococcus aureus in palatal mucosa			
	Group I (control group)	Group II	Group III	One way ANOVA
	$\:\stackrel{-}{X}\pm\:S.d$	$\:\stackrel{-}{X}\pm\:S.d$	$\:\stackrel{-}{X}\pm\:S.d$	F (*p*-value)
At insertion	8.67 ± 15.01	41 ± 12.77	23.33 ± 40.42	1.167 (0.373)
After 6 months	46.67 ± 25.15	33.33 ± 14.43	20 ± 34.64	0.784 (0.498)
After 12 months	38 ± 7.21	14 ± 24.25	15.33 ± 26.56	1.217 (0.360)
After 18 months	46 ± 4	12.67 ± 21.94	11.67 ± 20.21	3.794 (0.086)
F (p-value)	4.642 (0.153)	15.791 (0.050)	1.000 (0.423)	-------

A P value < 0.05 (*) indicates significance, and a P value < 0.001 (**) indicates high significance

No significant difference was found between different durations in the control group, with a p-value of 0.153. Additionally, no significant difference was found between different durations in Group 2, with a p-value of 0.050, and no significant difference was found between different durations in Group 3, with a p-value of 0.423. A comparison of the groups at each time point revealed no significant difference between all the groups, with a p-value of 0.373 at insertion; after 6 months, there was no significant difference between the groups, with a p-value of 0.498; after 12 months; however, no significant difference was found between all the groups (p-value of 0.360), and no significant difference was found between the groups (p-value of 0.086) after 18 months.

#### *Staphylococcus aureus* in the fitting surface of the denture

The standard deviation (SD) and mean values of the CFU of *Staphylococcus aureus* in the dentures throughout the duration for the studied groups and the comparisons between different durations in each group via repeated-measures ANOVA are illustrated in (Table 2).

**Table 2:** Analysis of CFU counts of *Staphylococcus aureus* in the fitting surface of the denture at different observation periods in the three groups

Duration	Staphylococcus aureus in the fitting surface of the denture			
	Group I (control group)	Group II	Group III	One way ANOVA
	$\:\stackrel{-}{X}\pm\:S.d$	$\:\stackrel{-}{X}\pm\:S.d$	$\:\stackrel{-}{X}\pm\:S.d$	F (*p*-value)
At insertion	0 ± 0	0 ± 0	0 ± 0	-------
After 6 months	28.33 ± 2.89	0 ± 0	0 ± 0	289 (0.000**)
After 12 months	34.33 ± 3.79	0 ± 0	0 ± 0	246.721 (0.000**)
After 18 months	37 ± 10.82	0 ± 0	0 ± 0	35.103 (0.000**)
F (p-value)	40.597 (0.023*)	-------	-------	-------

A P value < 0.05 (*) indicates significance, and a P value < 0.001 (**) indicates high significance

A significant difference was found between different durations in the control group, with a p-value of 0.023*as the highest count was after 18 months. A comparison of groups at each time point revealed highly significant differences between groups after 6 months, after 12 months, and after 18 months with a p-value of 0.000** as the control group showed the highest count and group II and group III showed zero counts all over the durations (Table 3).

**Table 3:** Shows the multiple comparison Tuckey test which is used to show the difference between each two groups

Duration	G I vs. G II	G I vs. G III	G II vs. G III
At insertion	0.000**	0.000**	1.000
After 6 months	0.000**	0.000**	1.000
After 12 months	0.000**	0.000**	1.000
After 18 months	0.001*	0.001*	1.000

There is a significant at P-value < 0.05 (*), and highly significant at P-value < 0.001 (**)

#### *Candida albicans* in the palatal mucosa

The standard deviation (SD) and mean values of the CFU of *Candida albicans* in the palate throughout the study duration for the studied groups and the comparisons between different durations in each group via repeated-measures ANOVA are shown in (Table 4).

**Table 4:** Analysis of CFU counts of *C. albicans* in the palatal mucosa at different observation periods in the three groups

Duration	Candida albicans in palatal mucosa			
	Group I (control group)	Group II	Group III	One way ANOVA
	$\:\stackrel{-}{X}\pm\:S.d$	$\:\stackrel{-}{X}\pm\:S.d$	$\:\stackrel{-}{X}\pm\:S.d$	F (*p*-value)
At insertion	19 ± 16.52	18.67 ± 16.29	23.33 ± 22.55	0.058 (0.944)
After 6 months	45.67 ± 14.01	8.33 ± 14.43	8.33 ± 14.43	6.821 (0.029*)
After 12 months	45.67 ± 19.14	0 ± 0	31.33 ± 6.03	12.191 (0.008*)
After 18 months	57 ± 13	0 ± 0	33 ± 12.12	23.326 (0.001*)
F (p-value)	2.879 (0.219)	2.285 (0.220)	4.586 (0.151)	-------

A P value < 0.05 (*) indicates significance, and a P value < 0.001 (**) indicates high significance

No significant difference was found between different durations in the control group, with a p-value of 0.219. Additionally, no significant difference was found between different durations in Group II (p-value of 0.220), and there was no significant difference between different durations in Group III, with a p-value of 0.151.

When groups at each duration were compared individually, no significant difference was found between groups (p-value of 0.944) at the time of insertion. After 6 months. Significant differences were found between groups after 6 months, after 12 months, and after 18 months with (p-values of 0.029*, 0.008*, and 0.001*), respectively as the control group showed the highest count and Group II was the lowest (Table 5).

**Table 5:** Shows the multiple comparison Tuckey test which is used to show the difference between each two groups

Duration	G I vs. G II	G I vs. G III	G II vs. G III
After 6 months	0.043*	0.043*	1.000
After 12 months	0.007*	0.349	0.037*
After 18 months	0.001*	0.064	0.018*

There is a significant at P-value < 0.05 (*), and highly significant at P-value < 0.001 (**)

#### *Candida albicans* in the fitting surface of the denture

The standard deviation (SD) and mean values of the CFUs of *Candida albicans* in the dentures throughout the duration for the studied groups and the comparisons between different durations in each group via repeated-measures ANOVA are shown in (Table 6).

**Table 6:** Analysis of CFU counts of *C. albicans* in the fitting surface of the denture at different observation periods in the three groups

Duration	Candida albicans in fitting surface of the denture			
	Group I (control group)	Group II	Group III	One way ANOVA
	$\:\stackrel{-}{X}\pm\:S.d$	$\:\stackrel{-}{X}\pm\:S.d$	$\:\stackrel{-}{X}\pm\:S.d$	F (*p*-value)
At insertion	0 ± 0	0 ± 0	0 ± 0	--------
After 6 months	32 ± 6.08	0 ± 0	8.67 ± 15.01	9.398 (0.014*)
After 12 months	43 ± 4.58	0 ± 0	26.67 ± 2.89	144.557 (0.000**)
After 18 months	51.33 ± 9.02	0 ± 0	36.67 ± 12.58	26.353 (0.001*)
F (p-value)	39.516 (0.021*)	--------	10.758 (0.066)	-------

A P value < 0.05 (*) indicates significance, and a P value < 0.001 (**) indicates high significance

A significant difference was found between different durations in the control group, with a p-value of 0.021*, whereas no significant difference was found between different durations in Group III, with a p-value of 0.066. In group II, CFUs of *candida* were zero at insertion and did not increase in any duration of follow-up. A significant difference between groups with a p-value of 0.014* after 6 months was detected as the control group showed the highest count (32 ± 6.08); a highly significant difference was detected between groups with a p-value of 0.000** after 12 months whereas the control group showed the highest count (43 ± 4.58), whereas after 18 months, a significant difference was detected between groups with a p-value of 0.001* as the control group showed the highest count (51.33 ± 9.02)(Table. 7).

**Table 7:** Shows the multiple comparison Tuckey test which is used to show the difference between each two groups

Duration	G I vs. G II	G I vs. G III	G II vs. G III
After 6 months	0.014*	0.051	0.529
After 12 months	0.000**	0.002*	0.000**
After 18 months	0.001*	0.191	0.006*

There is a significant at P-value < 0.05 (*), and highly significant at P-value < 0.001 (**)

### Patient satisfaction

#### Aesthetic

The standard deviation (SD) and mean values of the Aesthetics throughout the duration for the studied groups and the comparisons between different durations in each group are shown in (Table 8).

**Table 8:** Analysis of patient satisfaction at different observation periods in the three groups

Variables	Groups Duration	Group I(control group)	Group II	Group III	Kruskal-Wallis H (p-value)
		$\:\stackrel{-}{X}\pm\:S.d$	$\:\stackrel{-}{X}\pm\:S.d$	$\:\stackrel{-}{X}\pm\:S.d$	
Aesthetic	15 days after insertion	10 ± 0	10 ± 0	10 ± 0	0.001 (1.000)
	After 6 months	5.67 ± 1.16	9.67 ± 0.58	9.67 ± 0.58	6.000 (0.050)
	After 12 months	5.33 ± 0.58	9.67 ± 0.58	9.33 ± 1.16	5.969 (0.051)
	After 18 months	5 ± 0	9 ± 0	8.67 ± 0	7.000 (0.030*)
	Freidman (p-value)	7.909 (0.050)	6.333 (0.096)	7.000 (0.072)	-------
Speech	15 days after insertion	10 ± 0	10 ± 0	10 ± 0	0.001 (1.000)
	After 6 months	10 ± 0	10 ± 0	10 ± 0	0.001 (1.000)
	After 12 months	10 ± 0	10 ± 0	10 ± 0	0.001 (1.000)
	After 18 months	8.67 ± 0.58	9 ± 0	9.67 ± 0.58	4.413 (0.110)
	Freidman (p-value)	9.000 (0.057)	9.000 (0.057)	3.000(0.3292)	-------
Masticatory efficiency	15 days after insertion	10 ± 0	10 ± 0	10 ± 0	0.001 (1.000)
	After 6 months	9.33 ± 0.58	10 ± 0	9.67 ± 0.58	2.667 (0.264)
	After 12 months	8.33 ± 0.58	9.67 ± 0.58	9 ± 1	3.556 (0.169)
	After 18 months	6.67 ± 0.58	8.33 ± 0.58	9 ± 1	6.058 (0.048*)
	Freidman (p-value)	8.793 (0.032*)	9.143 (0.055)	3.375 (0.337)	-------
Hygiene	15 days after insertion	10 ± 0	10 ± 0	10 ± 0	0.001 (1.000)
	After 6 months	6.33 ± 2.31	10 ± 0	9.33 ± 1.16	5.603 (0.061)
	After 12 months	5.67 ± 1.16	10 ± 0	9.67 ± 0.58	6.788 (0.034*)
	After 18 months	5.33 ± 0.58	9 ± 0	9.33 ± 0.58	6.788 (0.034*)
	Freidman (p-value)	7.909 (0.048*)	9.000 (0.057)	3.000 (0.392)	-------
Comfort	15 days after insertion	10 ± 0	10 ± 0	10 ± 0	0.001 (1.000)
	After 6 months	6.33 ± 2.31	10 ± 0	10 ± 0	7.714 (0.021*)
	After 12 months	6.33 ± 1.53	9.67 ± 0.58	9.67 ± 0.58	5.945 (0.051)
	After 18 months	6 ± 1	9 ± 0	9.33 ± 1.16	5.843 (0.054)
	Freidman (p-value)	6.600 (0.086)	7.200 (0.066)	3.000 (0.392)	-------

P-value < 0.05 (*) means significant, and P-value < 0.001 (**) means highly significant

The comparison between groups revealed no significant difference between the studied groups at 15 days after insertion, after 6 months, and after 12 months, while a significant difference was found between the studied groups after 18 months, with a p-value of 0.030* whereas the group II showed the highest value and the best result (Table 9).

**Table 9:** Shows the multiple comparisons using the Mann-Whitney U test which was used to show the difference between the two groups regarding aesthetics

Duration	G I vs. G II	G I vs. G III	G II vs. G III
After 18 months	0.025*	0.034*	0.317

There is a significant at P-value < 0.05 (*), and highly significant at P-value < 0.001 (**)

To determine the effect of duration on each group, the Friedman test was used, which revealed no significant difference between the different durations in group I, with a p-value of 0.050.

, in group II, with a p-value of 0.096, or in group III, with a p-value of 0.072.

#### Speech

The standard deviation (SD) and mean values of the speech throughout the durations for the studied groups and the comparisons between different durations in each group are presented in (Table 8).

No significant difference was found between the studied groups at 15 days after insertion or 6, 12, and 18 months.

To determine the effect of duration on each group, the Friedman test was used, which revealed no significant difference between the different durations in group I, with a p-value of 0.057; in group II, with a p-value of 0.057; and in group III, with a p-value of 0.329.

#### Masticatory efficiency

The standard deviation (SD) and mean values of masticatory efficiency throughout the study duration for the studied groups and the comparisons between different durations in each group are shown in (Table 8).

No significant difference was found between the studied groups at 15 days after insertion, after 6 months, and after 12 months, since a significant difference was found between the studied groups after 18 months (*p* = 0.048*) whereas group III showed the highest value (Table 10).

**Table 10:** Shows the multiple comparisons using the Mann-Whitney U test which was used to show the difference between each two groups regarding masticatory efficiency

Duration	G I vs. G II	G I vs. G III	G II vs. G III
After 18 months	0.043*	0.046*	0.346

There is a significant at P-value < 0.05 (*), and highly significant at P-value < 0.001 (**)

To determine the effect of the duration in each group, the Friedman test was used, which revealed a significant difference between the different durations in group I, with a p-value of 0.032*, and no significant difference was found in the different durations in group II, with a p-value of 0.055, or in group III, with a p-value of 0.337.

#### Hygiene

The standard deviation (SD) and mean values of hygiene throughout the duration for the studied groups and the comparisons between different durations in each group are shown in (Table 8).

The comparison between groups revealed no significant difference between the studied groups at 15 days after insertion and after 6 months since a significant difference was found between the studied groups after 12 months and after 18 months, with p values of 0.034* and 0.034*, respectively whereas group II showed the highest value after 12 months and group III showed the highest value after 18 months (Table 11).

**Table 11:** Shows the multiple comparisons using the Mann-Whitney U test which was used to show the difference between the two groups regarding hygiene

Duration	G I vs. G II	G I vs. G III	G II vs. G III
After 12 months	0.034*	0.43*	0.317
After 18 months	0.034*	0.43*	0.317

There is a significant at P-value < 0.05 (*), and highly significant at P-value < 0.001 (**)

To determine the effect of duration on each group, the Friedman test was used, which revealed a significant difference between the different durations in group I, with a p-value of 0.048*; in group II, with a p-value of 0.057; and in group III, with a p-value of 0.392).

#### Comfort

The standard deviation (SD) and mean values of comfort throughout the study duration for the studied groups and the comparisons between different durations in each group are shown in (Table 8).

No significant difference was detected between the studied groups at 15 days after insertion, 12 months after insertion, and 18 months after insertion, since a significant difference was detected between the studied groups after 6 months, with a p-value of 0.021* whereas the control group showed the lowest value (Table 12).

**Table 12:** Shows the multiple comparisons using the Mann-Whitney U test which was used to show the difference between the two groups regarding comfort

Duration	G I vs. G II	G I vs. G III	G II vs. G III
After 6 months	0.034*	0.034*	1.000

There is a significant at P-value < 0.05 (*), and highly significant at P-value < 0.001 (**)

To determine the effect of duration on each group, the Friedman test was used, which revealed no significant difference between the different durations in group I, with a p-value of 0.086; in group II, with a p-value of 0.066; and in group III, with a p-value of 0.392.

## Discussion

To increase the quality of the denture base and decrease patient visits, the 3D printing method is used in prosthetic fabrication. However, conventional PMMA is still stronger than the 3D-printed resin used for denture bases [29]. 

Therefore, we planned in this study to add nano-ZrO_2_ and nano-TiO_2_ to the 3D-printed resin used for denture base, and the effects on microbial colonization and patient satisfaction after clinical use have not been fully explored.

Studies have suggested that zirconium dioxide can increase the quality, such as the microbial and mechanical properties, of 3D-printed resin used for denture bases [13]. Adding nano-TiO2 to 3D-printed denture base resin was shown to have antifungal activity against *Candida albicans* [29]. The nanocomposite is a biocompatible material with human gingival fibroblasts and is effectively used for 3D printing [29]. 

Nanoparticles with an average size of < 50 nm and a spherical shape were used, as it has been reported that the shape of the nanoparticles plays a vital role in their antibacterial performance, which shows that nanospheres have relatively close contact with the microorganism surface and that effective cellular uptake depends on adequately small nanoparticles [30]. 

The 3D printing resin was reinforced with 0.4% nano zirconium or 0.4% nano titanium by wt. The addition of 0.4% nanoTiO to a 3D-printed resin used for the denture base led to prominent antifungal activity. Concentrations of nanoparticles ranging from 0.4 to 2.5% successfully destroy Candida Scotti growth, and increasing the concentration results in nanoparticle aggregation and thus decreases the strength of the material [31]. 

The null hypothesis of our study was rejected with respect to microbial colonization and patient satisfaction.

In the present study, the number of colony-forming units of *C. albicans S. aureus* increased in group I (without nanoparticles) over time, which aligns with other studies that reported a significant increase in *C. albicans* adhesion in the 3D-printed resin group compared with the other groups (milled resins for denture base and conventional acrylic resin denture base) [32, 33]. 

3D-printed denture base materials have greater surface roughness than milled and heat-polymerized acrylic resin materials [34]. Another study revealed that microbial adhesion and bacterial colonization increase on rough denture surfaces, leading to the retention of areas for food debris that are difficult to remove, causing infections of the underlying tissues [35]. Additionally, a rough surface attracts more stains, which have the possibility of changing the material matrix, causing an external colorant stain [36]. 

The number of colony-forming units of *C. albicans* and *Staphylococcus aureus* in the palate decreased in group II (reinforced with nano ZrO_2_) over time, which agreed with the findings of another study that showed a significant decrease in the count of *C. albicans* in response to the addition of 0.5% nano-ZrO2 to the 3D-printed resin group compared with the other groups (conventional acrylic denture base resin and other groups of 3D-printed resins) [37]. Previous studies have shown that nano-ZrO_2_ has antibacterial activity [13, 37]. 

The number of colony-forming units of *C. albicans* and *Staphylococcus aureus* in the fitting surface of the denture was zero in group II (reinforced with nano ZrO_2_) in all durations. There is a shortage in clinical studies concerning to antimicrobial activity of nanoparticles but this result is in alignment with another in-vitro study that reported a decrease in the count of *C. albicans* and related it to the presence of specific nano-ZrO_2_ on the sample surface near the *C. albicans* cell membrane, which could convey antifungal abilities to the reinforced resin [38]. Additionally, Fareed et al. reported that adding nano-ZrO2 to 3D-printed resin for denture base leads to increased antifungal activity but depends on the concentration of nanoparticles [39]. 

This result was confirmed by another study in which nano-ZrO_2_ suppressed *Staphylococcus aureus* and *Escherichia coli* as bacteria and also *Aspergillus niger as* a fungus [40]. The high surface area and ability to prevent fungal growth by disrupting cell function are the main reasons for the antifungal activity of nanoZrO_2_. NPs inhibit the metabolic process of fungal cells by passing through their membrane and engaging in electrostatic attraction, hydrophobic contact, or van der Waals forces [38]. 

Studies have shown that the antimicrobial activity of nanoparticles is due to their ability to wrap around microbial cells, inhibit their regular budding process, and fragment microbial cells by creating pores that leak ions and alter the cell structure, leading to cell death [41]. 

For group III (reinforced with nano TiO_2_), the number of colony-forming units of *C. albicans* decreased until 6 months, and the number of colony-forming units of *S. aureus* decreased over time, as it was found that nano*-*TiO_2_ has antimicrobial activity against many strains, such as gram-positive and gram-negative bacteria, viruses and fungi. This may be due to the structure of the nanoparticles and their surface properties, such as their infrared reflectivity, noncontact antimicrobial effects, and hydrophilic surface activity [42]. 

The antifungal effect of nano-TiO_2_ in group III decreased at 12 months, which may be due to the variety of clinical conditions and many additional aspects, including the presence of saliva, dietary or parafunctional habits, neuromuscular and masticatory forces, and different cleansing techniques, which can affect the results [43] Additionally, the consumption of foods and beverages produces temperature changes that lead to a thermally dynamic medium in the intraoral environment. Therefore, degradation of the surface may occur due to thermal stress [44]. 

On the other hand, some studies reported that the antimicrobial activity of TiO2NPs when added to PMMA was achieved at a concentration of 5 wt%. However, the properties of the material decreased at this concentration [45, 46]. 

Compared with 3D-printed resin without additives, the addition of nano-ZrO2 and nano-TiO2 to 3D-printed denture base resin improved the aesthetics, speech, masticatory efficiency, hygiene, and comfort of patients.

This finding is in agreement with that of Cristache et al. [20]., who evaluated patient satisfaction before the insertion of 3D-printed dentures and then at 7 days, 12 months, and 18 months after the insertion of three groups: patients with maxillary complete dentures, patients with mandibular complete dentures, and patients with maxillary and mandibular complete dentures. Cristache et al. reported improvements in oral health-related quality of life, which may be due to good retention, denture base adaptation, stability, and minimal distortion at border seal areas of 3D-printed dentures [20]. 

Our result aligns with that of Altarazi et al., who reported an increase in the performance of a 3D-printed denture base reinforced with nano TiO_2_ (0.10 wt%) in comparison with 3D-printed resin without additives after aging in artificial saliva. The use of a nanocomposite denture base has been suggested to provide high-quality denture bases with prolonged service life after clinical use [47].

Concerning aesthetics, patients in the control group (without nanoparticles) reported greater stains on the denture base than patients in groups II (reinforced with nano ZrO_2_) and group III (reinforced with nano TiO_2_) did. This aligns with other studies in which PMMA was reinforced with different nanoparticles and the color stabilities of the samples were evaluated, and the results revealed greater color stability in the nano-ZrO_2_ group than in the other groups, suggesting that this approach could be used in trials to improve the color stability of the resin in the denture base [48]. 

The color change of the 3D-printed denture base resin was less clinically satisfactory than that of the conventional acrylic resin, which established greater color stability [49]. 

Another study revealed that 3D-printed maxillary complete dentures presented color changes after one year of use, which was less than the maximum acceptability threshold, according to the subjective assessment of the patient [50]. 

### Limitations 

Each patient has a different lifestyle and received one maxillary and mandibular 3D-printed complete denture for 18 months this may made changes in the result however we gave the same oral hygiene instructions to all patients so if the study was cross-over we think the result would be more accurate.

## Conclusion

- 1- Zirconium dioxide nanoparticles have antibacterial and antifungal effects until 18 months, while TiO2 nanoparticles have antifungal effects until 6 months and antibacterial effects until 18 months.
- 2- 3Dprinted denture base resin without additives has high bacterial adhesion and low patient satisfaction.
- 3- Adding ZrO_2_ and TiO_2_ nanoparticles to 3D-printed denture base resin improves the aesthetic, speech, masticatory efficiency, hygiene, and comfort of patients.

### Recommendation

- 1- Crossover studies are recommended to evaluate microbial colonization and patient satisfaction when different reinforced resins are used for maxillary denture bases with different nanoparticles.
- 2- Assessment of the staining of 3D-printed resin for denture bases reinforced with nanoparticles after long-term clinical use.

### The points of strength of this study

This study considered the first one to evaluate microbial colonization and patient satisfaction after clinical use of 3D printed denture base reinforced with nanoparticles and compare the effect of two types of nanoparticles.

## Electronic supplementary material

Below is the link to the electronic supplementary material.

Supplementary Material 1

## Data Availability

The datasets used and/or analysed during the current study are available from the corresponding author on reasonable request.

## Abbreviations

3D: 3Dimensions
S. aureus: Staphylococcus aureus
C. albicans: Candida albicans
ZrO2: Zirconium Dioxide
TiO2: Titanium Dioxide
CNC: computerized Numeric Control
NPs: Nanoparticles
STL: Standard Tessellation Language
LCD: Liquid Crystal Display
CFU: Colony-Forming Units

## References

[1] Goodacre CJ, Garbacea A, Naylor WP, Daher T, Marchack CB, Lowry J. CAD/CAM fabricated complete dentures: concepts and clinical methods of obtaining required morphological data. J Prosthet Dent. 2012;107:34–46. 22230914 10.1016/S0022-3913(12)60015-8

[2] Kattadiyil MT, Goodacre CJ, Baba NZ. CAD/CAM complete dentures: a review of two commercial fabrication systems. J Calif Dent Assoc. 2013;41:407–16. 23875432

[3] Kalberer N, Mehl A, Schimmel M, Müller F, Srinivasan M. CAD-CAM milled versus rapidly prototyped (3D-printed) complete dentures: an in vitro evaluation of trueness. J Prosthet Dent. 2019;121:637–43. 30711292 10.1016/j.prosdent.2018.09.001

[4] Huang SH, Liu P, Mokasdar A, Hou L. Additive manufacturing and its societal impact: a literature review. Int J Adv Manuf Technol. 2013;67:1191–203.

[5] Tahayeri A, Morgan M, Fugolin AP, Bompolaki D, Athirasala A, Ferracane JL. 3D printed versus conventionally cured provisional crown and Bridge dental materials. Dent Mater. 2018;34:192–200. 29110921 10.1016/j.dental.2017.10.003 PMC5801146

[6] Shim JS, Kim JE, Jeong SH, Choi YJ, Ryu JJ. Printing accuracy, mechanical properties, surface characteristics, and microbial adhesion of 3D-printed resins with various printing orientations. J Prosthet Dent. 2020;124:468–75. 31810611 10.1016/j.prosdent.2019.05.034

[7] Tymrak BM, Kreiger M, Pearce JM. Mechanical properties of components fabricated with open-source 3-D printers under realistic environmental conditions. Mater Des. 2014;58:242–6.

[8] Arslanov K. Oral microbiota changes in patients with complete dentures: a systematic review. Eur J Innov Nonform Educ. 2023;3:134–7.

[9] Aati S, Aneja S, Kassar M, Leung R, Nguyen A, Tran S, et al. Silver-loaded mesoporous silica nanoparticles enhanced the mechanical and antimicrobial properties of 3D-printed denture base resin. J Mech Behav Biomed Mater. 2022;134:1–12. 10.1016/j.jmbbm.2022.105421 36037709

[10] Kwan JC, Kwan N. Clinical application of PEEK as a provisional fixed dental prosthesis retained by reciprocated guide surfaces of healing abutments during dental implant treatment. Int J Oral Maxillofac Implants. 2021;36:581–92. 34115075 10.11607/jomi.8465

[11] Jordan J, Jacob KI, Tannenbaum R, Sharaf MA, Jasiuk I. Experimental trends in polymer nanocomposites—a review. Mater Sci Eng. 2005;393:1–11.

[12] Kaurani P, Hindocha A, Porwal A, Tambe A, Price C, Goel V. Effect of addition of metal oxide nanoparticles on the strength of Heat-Cured denture base resins: protocol for systematic review and Meta-Analysis of in vitro studies. JMIR Res Protoc. 2024;13:1–10. 10.2196/59999 PMC11464938 39321454

[13] Khattar A, Alghafli JA, Muheef MA, Alsalem AM, Al-Dubays MA, AlHussain HM. Antibiofilm activity of 3D-Printed nanocomposite resin: impact of ZrO2 nanoparticles. Nanomaterials. 2023;13:591–603. 36770550 10.3390/nano13030591 PMC9921268

[14] Gad MM, Al-Thobity AM, Shahin SY, Alsaqer BT, Ali AA. Inhibitory effect of zirconium oxide nanoparticles on Candida albicans adhesion to repaired polymethyl methacrylate denture bases and interim removable prostheses: a new approach for denture stomatitis prevention. Int J Nanomed. 2017;12:5409–19. 10.2147/IJN.S142857 PMC5546774 28814859

[15] Yasser AD, Fatah NA. The effect of the addition of zirconium nanoparticles on antifungal activity and some properties of soft denture lining material. J Baghdad Coll Dent. 2017;29:27–33.

[16] Ghahremani L, Shirkavand S, Akbari F, Sabzikari N. Tensile strength and impact strength of color-modified acrylic resin reinforced with titanium dioxide nanoparticles. J Clin Exp Dent. 2017;9:661–5. 10.4317/jced.53620 PMC5429478 28512543

[17] Alhotan A, Yates J, Zidan S, Haider J, Silikas N. Flexural strength and hardness of filler-reinforced PMMA targeted for denture base application. Materials. 2021;14:2659–72. 34069482 10.3390/ma14102659 PMC8159135

[18] Khalil MH, Sharabasy R, Abd Elsalam EB, Sharaf MY, Fawzy MF, Elhagali AF. Effect of various nanoparticles incorporated to 3D-Printed denture base resins on certain physicomechanical parameters. Twist. 2024;19:868–74.

[19] -Totu EE, Cristache CM, Vlasceanu G, Josceanu AM, Nechifor AC. On physical and chemical characteristics of Poly (methyl methacrylate) nanocomposites for dental applications. II Mater Plast. 2019;56:252–5.

[20] Cristache CM, Totu EE, Iorgulescu G, Pantazi A, Dorobantu D, Nechifor AC. Eighteen months follow-up with patient-centered outcomes assessment of complete dentures manufactured using a hybrid nanocomposite and additive CAD/CAM protocol. J Clin Med. 2020;9:324–43. 31979345 10.3390/jcm9020324 PMC7073708

[21] Ragheb N, Borg H. Antimicrobial effect of titanium oxide (Tio2) nano particles in completely edentulous patients. A randomized clinical trial. Adv Dent J. 2021;3:173–84.

[22] Abualsaud R, Aleraky DM, Akhtar S, Khan SQ, Gad MM. Antifungal activity of denture base resin containing nano zirconia: in vitro assessment of Candida albicans biofilm. Sci World J. 2021;2021:1–8. 10.1155/2021/5556413 PMC8352684 34381318

[23] Wood DJ. Techniques in complete denture technology. 1th ed. Wiley; 2012. P.7–24.

[24] Kortam S. Treatment outcomes of indirect versus direct digitally constructed complete dentures as compared to conventionally constructed complete dentures: Cross-Over clinical study. Egypt Dent J. 2022;68:1741–56.

[25] Grymak A, Badarneh A, Ma S, Choi JJE. Effect of various printing parameters on the accuracy (trueness and precision) of 3D-printed partial denture framework. J Mech Behav Biomed Mater. 2023;140:1–10. 10.1016/j.jmbbm.2023.105688 36753847

[26] El Naggar SM, Maged A, Elawady AF, Mahmoud TA. Masticatory performance and patient satisfaction of metal-reinforced and CAD/CAM-fabricated acrylic resin mandibular complete dentures. J Arab Soc Med Res. 2023;18:35–42.

[27] Kholief DM, Kabeel SM. Assessment of microbial adherence on conventional and CAD/CAM complete denture. Al-Azhar Dents J Girls. 2019;6:9–17.

[28] Rani S, Dhawan P, Saxena V. A randomized clinical trial comparing retention of complete dentures and oral health quality of life of patients with conventional and bioelectric impressions. J Oral Biol Craniofac Res. 2025;15:103–7. 39810834 10.1016/j.jobcr.2024.12.006 PMC11732451

[29] Altarazi A, Jadaan L, McBain AJ, Haider J, Kushnerev E, Yates JM. 3D-printed nanocomposite denture base resin: the effect of incorporating TiO2 nanoparticles on the growth of Candida albicans. J Prosthodont. 2024;33:25–34. 37837403 10.1111/jopr.13784

[30] Menichetti A, Mavridi-Printezi A, Mordini D, Montalti M. Effect of size, shape and surface functionalization on the antibacterial activity of silver nanoparticles. J Funct Biomater. 2023;14:244–64. 37233354 10.3390/jfb14050244 PMC10219039

[31] Totu EE, Nechifor AC, Nechifor G, Aboul-Enein HY, Cristache CM. Poly (methyl methacrylate) with TiO2 nanoparticles inclusion for stereolithography complete denture manufacturing– the future in dental care for elderly edentulous patients? J Dent. 2017;59:68–77. 28223199 10.1016/j.jdent.2017.02.012

[32] Di Fiore A, Meneghello R, Brun P, Rosso S, Gattazzo A, Stellini E. Comparison of the flexural and surface properties of milled, 3D-printed, and heat polymerized PMMA resins for denture bases: an in vitro study. J Prosthodont Res. 2022;66:502–8. 34853238 10.2186/jpr.JPR_D_21_00116

[33] Meirowitz A, Rahmanov A, Shlomo E, Zelikman H, Dolev E, Sterer N. Effect of denture base fabrication technique on Candida albicans adhesion in vitro. Materials. 2021;14:221–8. 33466383 10.3390/ma14010221 PMC7795816

[34] Ozden YE, Atali PY, Kayahan ZO. Impact of fabrication techniques and Polishing procedures on surface roughness of denture base resins. J Vis Exp. 2025;215:67844–55. 10.3791/67844 39895617

[35] Berger JC, Driscoll CF, Romberg E, Luo Q, Thompson G. Surface roughness of denture base acrylic resins after processing and after Polishing. J Prosthodont. 2006;15:180–6. 16681500 10.1111/j.1532-849X.2006.00098.x

[36] Alqanas SS, Alfuhaid RA, Alghamdi SF, Al-Qarni FD, Gad MM. Effect of denture cleansers on the surface properties and color stability of 3D printed denture base materials. J Dent. 2022;120:1–7. 10.1016/j.jdent.2022.104089 35271942

[37] Gowri S, Gandhi R, Rajiv Sundrarajan M. Structural, optical, antibacterial and antifungal properties of zirconia nanoparticles by biobased protocol. J Mater Sci Technol. 2014;30:782–90.

[38] -Ahmad N, Jafri Z, Khan ZH. Evaluation of nanomaterials to prevent oral candidiasis in PMMA-based denture-wearing patients. A systematic analysis. J Oral Biol Craniofac Res. 2020;10:189–93. 32373449 10.1016/j.jobcr.2020.04.012 PMC7193251

[39] Fareed Al-Sammraaie M, A Fatalla A. The effect of ZrO2 nanoparticles addition on Candida adherence and tensile strength of 3D printed denture base resin. J Nanostruct. 2023;13:544–52.

[40] Jangra SL, Stalin K, Dilbaghi N, Kumar S, Tawale J, Singh SP. Antimicrobial activity of zirconia (ZrO2) nanoparticles and zirconium complexes. J Nanosci Nanotechnol. 2012;12:7105–12. 23035440 10.1166/jnn.2012.6574

[41] Rehman S, Asiri SM, Khan FA, Jermy BR, Khan H, Akhtar S. Biocompatible Tin oxide nanoparticles: synthesis, antibacterial, anticandidal and cytotoxic activities. ChemistrySelect. 2019;4:4013–7.

[42] Aziz HK. TiO2-nanofillers affect some properties of highly-impact resin using different processing techniques. Open Dent J. 2018;12:202–12. 29643946 10.2174/1874210601812010202 PMC5872201

[43] Azmy E, Al-Kholy MRZ, Al-Thobity AM, Gad MM, Helal MA. Comparative effect of incorporation of ZrO2, TiO2, and SiO2 nanoparticles on the strength and surface properties of PMMA denture base material: an in vitro study. Int J Biomater. 2022;2022:1–10. 10.1155/2022/5856545 PMC9072016 35528846

[44] Çakmak G, Molinero-Mourelle P, De Paula MS, Akay C, Cuellar AR, Donmez MB. Surface roughness and color stability of 3D-printed denture base materials after simulated brushing and thermocycling. Materials. 2022;15:6441–50. 36143757 10.3390/ma15186441 PMC9503686

[45] Tsuji M, Ueda T, Sawaki K, Kawaguchi M, Sakurai K. Biocompatibility of a titanium dioxide-coating method for denture base acrylic resin. Gerodontology. 2016;33:539–44. 26223290 10.1111/ger.12204

[46] Cheng Y, Sakai T, Moroi R, Nakagawa M, Sakai H, Ogata T, et al. The self-cleaning ability of a photocatalyst-containing denture base material. Dent Mater J. 2008;27:179–86. 18540390 10.4012/dmj.27.179

[47] Altarazi A, Haider J, Alhotan A, Silikas N, Devlin H. 3D printed denture base material: the effect of incorporating TiO2 nanoparticles and artificial aging on the physical and mechanical properties. Dent Mater. 2023;39:1122–36. 37839997 10.1016/j.dental.2023.10.005

[48] Azmy E, Al-Kholy MRZ, Gad MM, Al-Thobity AM, Emam A-NM, Helal MA. Influence of different beverages on the color stability of nanocomposite denture base materials. Int J Dent. 2021;2021:1–9. 10.1155/2021/5861848 PMC8601796 34804165

[49] Al-Ameri A, Alothman OY, Alsadon O, Bangalore D. An In-Vitro evaluation of strength, hardness, and color stability of Heat-Polymerized and 3D-Printed denture base polymers after aging. Polymers. 2025;17:288–305. 39940491 10.3390/polym17030288 PMC11820030

[50] Cristache CM, Oancea L, Didilescu AC, Burlibasa M, Totu EE. Color changes and stainability of complete dentures manufactured using PMMA-TiO2 nanocomposite and 3D printing technology year evaluation. Rev Chim. 2018;69:463–8.

